# Bullying and Its Associated Individual, Peer, Family and School Factors: Evidence from Malaysian National Secondary School Students

**DOI:** 10.3390/ijerph18137208

**Published:** 2021-07-05

**Authors:** Vikneswaran Sabramani, Idayu Badilla Idris, Halim Ismail, Thiyagar Nadarajaw, Ezarina Zakaria, Mohammad Rahim Kamaluddin

**Affiliations:** 1 Department of Community Health, Faculty of Medicine, Universiti Kebangasaan Malaysia Medical Center, 56000 Kuala Lumpur, Selangor, Malaysia; dr_idayu@ppukm.ukm.edu.my (I.B.I.); halimismail@ppukm.ukm.edu.my (H.I.); 2 SV Care Medic Sdn Bhd, No. 58 Jalan PP 16/2, Perdana Industrial Park, Taman Putra Perdana, Puchong 47130, Selangor, Malaysia; 3 Department of Pediatrics, Hospital Sultanah Bahiyah, Alor Setar 05460, Kedah, Malaysia; thiyagarnadarajaw@yahoo.com; 4 Centre for Research in Psychology and Human Well-Being, Faculty of Social Sciences and Humanities, Universiti Kebangsaan Malaysia, Bangi 43600, Selangor, Malaysia; ezaz@ukm.edu.my

**Keywords:** bullying behaviour, individual factors, peer factors, family factors, school factors

## Abstract

Adolescents involved in bullying can be at risk of developing behavioural problems, physical health problems and suicidal ideation. In view of this, a quantitative research design using a cross-sectional study was conducted to determine the prevalence of bullying and associated individual, peer, family and school factors. The study involved 4469 Malaysian public-school students who made up the response rate of 89.4%. The students were selected using a randomized multilevel sampling method. The study found that 79.1% of student respondents were involved in bullying as perpetrators (14.4%), victims (16.3%), or bully–victims (48.4%). In a multivariate analysis, the individual domain showed a significant association between students’ bullying involvement and age (OR = 1.38; 95% CI 1.12–1.70), gender (OR = 1.73; 95% CI 1.47–0.91), ethnicity (OR = 0.66; 95% CI 0.47–0.91), duration of time spent on social media during the weekends (OR = 1.43; 95% CI 1.09–1.87) and psychological distress level (OR = 2.55; 95% CI 1.94–3.34). In the peer domain, the significantly associated factors were the number of peers (OR = 0.69; 95% CI 0.56–0.86) and frequency of quarrels or fights with peers (OR = 2.12; 95% CI 1.24–3.26). Among the items in the school domain, the significantly associated factors were students being mischievous in classrooms (OR = 1.52; 95% CI 1.06–2.06), student’s affection towards their teachers (OR = 1.53; 95% CI 1.06–2.20), frequency of appraisal from teachers (OR = 1.49; 95% CI 1.16–1.94), frequency of friends being helpful in classrooms (OR = 1.92; 95% CI 1.09–3.38) and frequency of deliberately skipping class (OR = 2.91; 95% CI 2.90–1.72). As a conclusion, the study revealed high levels and widespread bullying involvement among students in Malaysia. As such, timely bullying preventions and interventions are essential, especially in terms of enhancing their mental health capacity, which substantially influences the reduction in the prevalence rates of bullying involvement among students in Malaysia.

## 1. Introduction

Bullying is a serious public health issue, requiring attention not only because of the effects it has on people but its tendency to continue even in the presence of bystanders [1]. In the 21st century, bullying has become an increasingly common phenomenon with 5% to 15% of people being the victim of bullying reported worldwide, among which some cases even lead to deaths [2]. Moreover, there has been an increase in cases of deaths by suicide and use of aggression among adolescents due to bullying in schools [3]. The involvement of adolescents in bullying is highly apparent and affects almost half of them all over the world [4].

Bullying is characterized as a set of aggressive behaviours [5], systematic abuse of power and a form of peer abuse [6], involving three primary elements, namely, repetition, harm and power imbalance [7]. Therefore, an individual is considered a victim of bullying when and if they have been continuously and repetitively exposed to harmful or negative behaviour, enacted by another individual or multiple other individuals, without having the power to defend themselves [8]. The power imbalance that exists between the individuals will likely put the weaker individual in harm’s way [8].

It has been documented via past literature that victims of bullying have a probability of experiencing problems related to health, mental well-being and academic performance. They are also found to be more likely to experience depression, anxiety and low self-esteem, compared to adolescents who have not been victims of bullying [5,9,10]. Additionally, research has identified that depression, anxiety and low self-esteem are also precursors to bullying and not just outcomes of it [11,12]. Therefore, adolescent students who are involved in bullying are more likely to experience depression, anxiety and low self-esteem as compared to their peers. Past studies have also identified several physical health problems that are prominent among adolescents who are involved in bullying. It was found that bullied teens were more likely to experience abdominal pain, problems with sleep, nonspecific headaches, muscle strain, extreme fatigue and suppressed appetites [11]. In a meta-analysis conducted by [13], it was found that children who were bullied were significantly more at risk of experiencing psychosomatic problems as opposed to their peers who remain uninvolved in bullying.

In addition, there was also proof that those involved in bullying had lower academic excellence. Moreover, teenagers involved in bullying are more likely to be frequently absent from school [14], have a higher rate of truancy [15,16], dislike school and score lower in exams and standardized tests [17]. Meta-analytic research that involved the analysis of 33 journal articles [18] found that there was a negative correlation between involvement in bullying and academic performance, which was measured based on grades achieved, achievement scores or teachers’ acknowledgement of their success.

Generally, many factors are associated with adolescents involved in bullying, ranging from individual factors to peer influence, family and school factors [19]. From the aspect of individual factors, research has proven that individuals who have difficulties with social adjustment are at risk of being involved in bullying [20,21]. In addition, peer influence, familial factors, school environment and community influences are highly likely to act as risk factors that lead an individual to be involved in bullying [22]. Therefore, to prevent student involvement in bullying, proper and clear preventative policies and active participation of relevant and responsible parties is required. Early adolescence is the ideal time to encourage anti-bullying sentiments, as it becomes harder and more costly to fix or undo these patterns of negative behaviour if they have participated in it for too long. Therefore, identifying the associated risk factors that lead an individual to engage in bullying behaviours will aid in prevention strategies, as well as possibly steer high-risk individuals away from being involved in bullying [23]. This can also go on to positively influence and reduce physical and mental problems that adolescents face in relation to their exposure to bullying.

### The Current Study

The issue of bullying in Malaysian schools is a cause for concern to responsible authorities, as well as the public, so much so that component of bullying has been included in a national-level study carried out by the Malaysian Institute of Public Health. The National Health and Morbidity survey, carried out in the year 2012, studied the prevalence rates of victims of bullying, the number of days they were bullied out of the past 30 days and the most common method that was used to bully the respondents. In conclusion, the survey identified that the prevalence rate of victimization was 17.9%. However, the survey only measured bullying from the perspective of victims alone [24].

In order to obtain a more accurate understanding of the actual situation of bullying within the country, it is important to not only identify the victims, but also the bullies and the individuals who play both the role of the victim and that of the bully. Additionally, there are no studies that link various risk factors of bullying participation among students. Studies that elaborate on bullying activities in Malaysia are also fairly limited in comparison to other countries. Therefore, the true weight of the situation within the nation cannot be determined, limiting the opportunities to formulate policies that would help prevent bullying in the country. Preventative strategies like those used internationally might not be suitable to be used in Malaysia, as the measures used have not been validated locally and, hence, could produce results that do not align with the bullying situation in the country due to cross-cultural differences.

Based on the studies by [25], there is a clear increase in the usage of handphones, as well as the access to the internet, among Malaysian adolescents, making the threat of cyberbullying inevitable. Moreover, it opens up the possibility of abuse by individuals from older age groups, having taken into consideration the fact that strength is unidentifiable online and there is no physical contact with the victim. The lack of literature pertaining to cyberbullying within the country has afforded the researcher the opportunity to identify the current prevalence of cyberbullying cases in Malaysia. Additionally, the study aimed to identify the prevalence of adolescent involvement in bullying, as well as the associated factors such as individual, peer, family and school factors, which contribute to adolescent engagement in bullying in Malaysian secondary schools. As such, it is hypothesised, there is an association between individual, family, peer and school factors with involvement in bullying among National secondary school students in Malaysia.

## 2. Materials and Methods

The present research adopted a quantitative research design using a cross-sectional research approach. Selected students were required to complete self-administered questionnaires, which were then collected and used to identify and assess their involvement in bullying scenarios. Data were collected via the self-administered questionnaires that were completed by selected students after informed consent was obtained from parents of participating students. Domains of the questionnaires used in the current study were considered reliable and valid, as suggested by Vikneswaran, Ismail, Idris, Kamaluddin, Ratnakrisna and Wani [26].

A total of 55 items were used to measure 5 different domains of dependent variables; 21 items measured the individual factors, 4 items measured peer factors, 20 items measured family factors and 10 items measured school factors. Following that, 25 items measured factors relating to the independent variables. Out of the 25 items, 13 items addressed bullying, while the remaining 12 evaluated victimization. Overall, students were required to answer 80 items, which were obtained and adapted from various sources.

The study was conducted in the year 2017 for duration of 6 months. It was carried out in public secondary schools within Peninsular Malaysia, Sabah, Sarawak and the Federal Territory of Labuan, after having obtained approval from the Research Ethics Committee of the National University of Malaysia and the Malaysian Education Ministry. Before the survey was conducted, written informed consent forms were obtained from parents whose children had participated in the study.

### 2.1. Background of Area of Research & Population

Malaysia is a country that is 329,847 per square kilometres wide, placed in Southeast Asia. It is made up of 13 states and 3 Federal Territories and citizens of various cultures, with Islam as the official religion as deemed by the country’s constitution, which also states that the freedom for citizens of other religions to freely practice their religion will be protected by the law. In the year 2016, 31.7 million made up the population of Malaysia, with 22.6 million of them in Peninsular Malaysia and the rest from the remaining states, namely, Sabah, Sarawak and the Federal Territory of Labuan [27].

In general, secondary education in Malaysia is 5 years long and is known as Forms 1 to 5. Form 1 to Form 3 students are categorized as “Lower Secondary” students, while students in Form 4 and 5, belong to the “Upper Secondary” category. In total, there are about 2,344,891 secondary school students in the country. 66.0% of this total number is made up of male students, while female students make up the remaining 34%. Out of all the schools in the nation, 2414 of them are managed by the government while 75 schools are either private or international schools [27]. Having taken into consideration the time constraints in carrying out the study, ethics approval from relevant authorities, as well as obtaining access to school premises, this study was conducted in public schools.

### 2.2. Sampling Method

A list of secondary schools in Malaysia was obtained from the Malaysian Ministry of Education and a multistage sampling method was used in this study to obtain the final sample. A proportional sampling method was used to select certain secondary schools from the 13 states and 3 federal territories of Malaysia, based on a few set criteria. The schools were selected as potential participants of the study. The criteria that the schools had to meet were, admission levels exceeding 2000 students and the presence of students of both genders (i.e., male and female) and after applying the criteria to the selection process, a total of 84 schools were shortlisted, following which a random sampling method was employed to further narrow the sample down into a feasible sample of 24 schools which represented all the states within the country. The final group of schools which were selected made up 1/3rd the total number of schools that met the criteria set.

Moving forward, a quota sampling method was utilized to select the classes from which participants will be selected. These classes were chosen by the guidance counsellors of each school, based on the academic performance of the students within the classrooms. Therefore, each classroom had students who fell into high, intermediate and low achieving strata. Finally, a convenience sampling method was used to select the final group of potential participants. The students were selected using a randomization table. 5000 informed consent forms were distributed to the chosen students and a total of 4469 students had confirmed their participation, giving the researchers a response rate of 89.38%. Students involved in the study were required to be Malaysian students, whose parents have provided written informed consent allowing their children to partake in the study.

### 2.3. Measures

#### 2.3.1. Section A: Demographic Details

Demographic details students were required to provide were their date of birth, age, gender, race, weight, height and email address.

#### 2.3.2. Section B: Individual, Peer, Family and School Factors

Individual factors measured were related to media usage habits and level of psychological distress. Peer factors, on the other hand, addressed the number of friends an individual has overall, how readily friends were willing to provide support and the frequencies of time spent together as well as quarrels or arguments between the respondent and their friends. Family factors aimed to measure characteristics of parenting attitudes, as well as those of siblings and other members of the household. School factors that were investigated include school environment, teachers’ attitudes towards the respondent, classroom characteristics and a sense of belonging in school.

#### 2.3.3. Section C: Kessler Psychological Distress Scale (K10)

This section contained 10 items that assessed the frequency of distress symptoms experienced by the respondent. Frequencies were assessed using a 5-point Likert scale, where 1 = never, 2 = rarely, 3 = sometimes, 4 = frequently and 5 = always.

#### 2.3.4. Section D: Malaysian Bullying Questionnaire

Details obtained via this section were related to bullying activities. The items aimed to identify the roles of individuals (i.e., bully, victim, bully-victim), as well as the types of bullying they were involved in (i.e., physical, verbal, relational and cyber-based bullying).

### 2.4. Data Analyses

Data analysis was carried out using the 23.0 version of the Statistical Package for Social Sciences (SPSS). The descriptive and statistical analyses were computed using this software. Levels of significance were *p* < 0.05, with a 95% confidence interval. The continuous variable was simplified using means and standard deviations. All variables were deemed necessary and represented as numbers (n) or percentages (%). Then, a simple logistic regression analysis was carried out as a bivariate analysis to distinguish the statistical differences between those categorical variables. Finally, a multiple logistic regression analysis was implemented to identify the relationship between the data collected and the factors investigated, covariate adjustment, or confounders and to identify important factors that influence an adolescent student’s involvement in bullying.

## 3. Results

### 3.1. Prevalence of Bullying

Table 1 presents the findings regarding the distribution of Malaysian public secondary school students’ involvement in bullying. Out of 4469 students, only 20.9% had stated that they were not involved in bullying incidences, while the majority (79.1%) had admitted to having been involved in bullying, as either a bully (14.4%), victim (16.3%), or bully–victim (48.4%). When further probed about the types of bullying they had been involved in, it was found that most bullies had resorted to verbal bullying (55.6%), followed by physical bullying (36.7%), relational (11.6%) and, lastly, cyberbullying (5.3%), which was the least common type of bullying a bully would engage in. From the victims’ perspective, the most common type of bullying they had experienced was also in a similar order, whereby verbal bullying was found to be the most common (51.2%), followed by relational bullying (40.4%), physical bullying (27.3%) and cyberbullying (13.1%).

### 3.2. Distribution of Individual Factors

Dependent variables, as well as risk factors, were categorized into various domains, namely individual factors, peer factors, family factors and school factors. A total of 1374 students, who make up 30.7% of the respondents, were 13 years old. The 14-year-old students, on the other hand, made up the majority of the respondents, with 1652 (37.0%) students and the remaining 1443 (32.3%) respondents were 16-year-olds.

In addition to the demographic details of the respondents, their social media behaviours, as well as other relevant factors, were measured. An overwhelming majority (80.2%) of the respondents stated that they own a personal handphone, while only 19.8% had stated otherwise. Similarly, only a minority of respondents (33.5%) had access to no other technological devices (i.e., iPad, iPod, tablet, personal computer), whereas the remaining majority (66.5%) owned such devices. Most students (67.1%) access social media for a few minutes up to about 4 h a day on a weekday and this number decreases to 60.9% on weekends. A total of 3008 students who made up 67.3% of the respondents access the internet for a few minutes up to 4 h a day on weekdays and this number is reduced to 2563 students (57.3%) on weekends. More than half of the students (56.4%) access the internet on a daily basis. A total of 75.7% experience some form of psychological distress, with most of them experiencing slight distress (31.2%), followed by moderate levels of distress (25.5%) and, lastly, severe distress (19.0%).

### 3.3. Distribution of Peer Factors

The distribution of the characteristics of peer factors is displayed in Table 2. Most adolescents consider themselves as having many friends (77.7%), followed by a few friends (15.5%), while a small fraction of them (5.9%) said they do not have many friends at all and an even smaller minority of 0.9% stated that they have no friends at all. A total of 4109 (91.9%) of students had close friends and almost half of them rarely argue with or fight each other (49.7%). One-third of them (31.1%) had admitted to sometimes quarrelling or fighting with their friend and, lastly, 4.9% had stated that they frequently quarrel with their friends. Regarding time spent with their friends, most (59.2%) stated that they sometimes spend time with friends, whereas 30.8% had claimed that they rarely spend time with friends.

### 3.4. Distribution of Family Factors

The next set of risk factors addressed were categorized as family factors, which are displayed below, in Table 3. They address factors that describe the characteristics of the parents and student respondents. The data pertaining to the education level of the respondents’ parents show that the majority of mothers and fathers had, at the very least, completed their secondary school education, with 63.4% of mothers and 60.2% of fathers having completed their secondary school education. However, a small percentage of mothers (6.8%) and fathers (8.1%) were unable to access any form of formal education.

With regards to their career background, 50.5% of fathers and 28.6% of mothers are non-professionals, whereas 37.0% and 27.2% of fathers and mothers, respectively, are professionals and the remaining small percentage of them are retirees. Additionally, 41.4% of mothers were found to have fallen into the “other” category, which could mean that they are either unemployed or involved in businesses. When both parents’ incomes were combined, the majority of household incomes were categorized between RM 1000 per month to RM 5000 per month (69.9%), while a small percentage of them earned more than RM 10,000 per month (9.4%). A total of 90.9% of respondents’ parents remain married, while 6.2% were divorced and 12.9% were included in the “Other” category. On average, 51.0% of fathers spent around 1 to 6 h with their children daily, while only a small percentage of them spent more than 6 h. Unlike fathers, however, most mothers (37.3%) spent more than 10 h on average with their children. This is followed by 34.4% of mothers who spent 1 to 6 h with their children and 22.8% who spent around 7 to 10 h. Only 17.3% of students had stated that their parents helped with homework.

In terms of parenting styles in further detail and the respondents’ relationship with their parents, the majority of them (70.4%) had stated that their parents were usually aware of the whereabouts of the children. Moreover, 71.5% of parents were categorized as being always and sometimes over-protective of their children and 88.8% of respondents perceived their parents as being immature in dealing with their children. Students also stated that their parents rarely quarrel or fight with each other (46.4%). The respondents also claimed that they rarely quarrel or fight with their parents (56.4%). A total of 60.0% of the respondents had more than four household family members and a majority of them (93.3%) had siblings. Out of the percentage of respondents that identified as having siblings, 64.8% of them stated that they have one to four siblings who were either older or younger than them. When compared, there was a higher percentage of students who always or sometimes share their feelings with their mother (68.2%) in contrast to their father (41.7%).

### 3.5. Distribution of School Factors

Factors related to school were part of another dimension addressed and studied. Table 4 presents the data obtained and the variables categorized as school factors. Among factors studied when addressing a school’s environment, was a student’s interest in attending school, as well as their love and affection towards their school. It was found that 87.9% of them were interested in attending school daily and 85.8% of them stated that they do feel love and affection towards their school.

The majority of the students (71.5%) believe that they have at least one individual present in school with whom they can share their feelings. In line with that, only about 9.1% of students admitted to not having respect or affection towards their teachers. Almost 79.0% of students “always” and “sometimes” received appraisal from their teachers, have friends who are very helpful in class and have a strong sense of belonging in school. A total of 3962 students denied being mischievous or troublemakers in class and a majority of them (66.2%) had never intentionally skipped class, while a small number of students (6.4%) had admitted to doing so around four times to date. Only 22.5% of students had never been truant throughout their schooling years and among them, most (68.6%) had been truant for at least a day, up to 15 days throughout their schooling years.

### 3.6. Univariate Variable Analyses

Simple logistic regression was used to identify the significant relationship between dependent variables and students’ involvement in bullying behaviour. Table 5 shows that the level of psychological distress of students is significantly related to bullying behaviour, whereby those who displayed severe distress were 3.54 (95% CI = 2.76–4.55) times more likely to engage in bullying behaviours, with male students being 85.0% more at risk of being involved in bullying. Additionally, students categorized as having “unsatisfactory” academic performance were 1.67 (95% CI = 1.18–2.36) times more prone to engage in bullying. In terms of ethnicity, however, Chinese adolescents were 0.59 (95% CI = 0.43–0.83) times less likely to be involved in bullying.

Those who used the internet increased their chances of being involved in bullying by 50.0% in comparison to those who do not and the students who spent 4 h or more on weekdays and weekends were 2.10 (95% CI = 1.53–2.67) and 2.25 (95% CI = 1.69–2.90) times more likely to be involved in bullying, respectively. Therefore, time spent on the internet was significantly related to bullying involvement. Students who accessed social media via the internet for over 4 h during weekdays and the weekend, were 2.10 (95% CI = 1.57–2.80) and 2.56 (95% CI = 1.53–2.69) times more prone to being involved in bullying, respectively.

Table 6 shows that having a lot of friends and close friends significantly reduces a student’s chance of being involved in bullying by 0.44 (95% CI = 0.11–0.85) and 0.72 (95% CI = 1.533–2.69) times, respectively. Conversely, students who frequently had quarrels and fights with their friends and always spent time with their friends, were 3.86 (95% CI = 0.11–0.85) and 2.10 (95% CI = 1.53–2.69) times more likely to be involved in bullying, respectively.

Table 7 shows that characteristics of parents, father’s level of education and career level are significantly related to the respondent’s bullying involvement. Additionally, students whose parents earn RM 5000 to RM 10,000 have a 1.73 (95% CI = 1.30–2.38) higher probability of being involved in bullying.

Upon further inspection of the characteristics of parents in terms of their interaction with their children, it was identified that students with parents who rarely helped their child (ren) with homework were 1.46 (95% CI = 1.16–1.82) times more likely to be involved in bullying. In addition, parents who are not aware of their children’s whereabouts, as well as those who always argue with each other, increase the risk of the child’s (i.e., the respondent) bullying involvement by 114.0% and 83.0%, respectively. Students who always argue with their parents and those who have overly protective parents, were 2.04 (95% CI = 1.26–3.30) and 1.40 (95% CI = 1.06–1.86) times more at risk to be involved in bullying than others, respectively.

Table 8 shows the relationship between school factors and bullying involvement. It shows that students who have very little interest in attending school, lack a sense of love and affection towards their school and do not have at least one individual they can share their feelings with within the school are at significant risk of being involved in bullying. Those who had little interest in attending school were 3.52 (95% CI = 1.53–2.61) times more significantly likely to be involved in bullying.

When evaluating their interactions with teachers, it was found that students who lacked affection towards their teacher and “rarely” or “never” received appraisals by their teachers were more likely to be at significant risk of bullying involvement. Within the classroom, on the other hand, students who identified as being mischievous or troublemakers and those who rarely had helpful friends within the classroom, both, respectively, were 2.76 (95% CI = 2.03–3.75) and 3.48 (95% CI = 2.06–5.89) times higher likelihood of being involved in situations of bullying. The implications of this result can be observed in the relationship between bullying involvement and a student’s relationship with the school as well. Those who rarely or never felt a sense of belonging in school had a high frequency of skipping classes and a school truancy frequency of almost a month in the past years, were more likely to be involved in bullying.

Other than significant dependent variables, all important dependent variables that were clinically important but were not significant in this study were also included in the regression model. In the beginning, “Backward LR” and “Forward LR” were first used to determine the variables to be used. After careful evaluation of the obtained results, the findings obtained from “Backward LR” was accepted as a stable model was observed. Other than that, model preliminary main effect model was checked for multicollinearity using linear regression analysis (Table 9) and no collinearity was reported. The regression model was statistically stable.

On checking the assumptions and outliers, Hosmer and Lemeshow test (Table 10) was performed to measure the goodness-of-fit and Classification Table was checked (Table 11). Upon obtaining the insignificant ‘p value’ result, it was concluded that the model and data fitted well to the logistic model and the dataset relationship pattern was not significantly different from the theoretical logistic model. Moreover 80.0% of cases were predicted correctly whether the students were involved in bullying or not.

In order to test outliers, Cook’s influential statistics was carried out. Data points above 1.0 were assumed to be outliers. In the data collected through and for the study, there were no values above 1.0, as the maximum value obtained was 0.78. Therefore, there were no outliers.

“Backward LR” was used (all variables were included in multiple logistics regression). There were no collinearities reported. The classification table (percentage of accurate classification = 80%), Table 12 shows factors that relate to bullying involvement. Under the domain that discussed individual factors, it was found that adolescents aged 13 had a 1.38 (95% CI = 1.12–1.70) times higher possibility of being involved in bullying, compared to individuals who were 16 years old. Aside from that, being male increased an individual’s risk of being involved in bullying by 73.0%, in comparison to female students. Moreover, in terms of ethnicity, Chinese students were 55.0% less likely to be involved in bullying, followed by Indian students who had a 44.0% lower chance and Malays whose chances reduced by 34.0%, in comparison to the adolescents from other ethnicities.

When studying patterns of behaviour associated with social media access, it was found that individuals who spent 1 to 4 h a day during weekends had a 1.43 (95% CI = 1.09–1.87) times higher likelihood of bullying involvement. This is followed by those who spent over hours a day on a weekend, who increased their chances of bullying involvement by 1.28 (95% CI = 1.69–3.10) times. Severe psychological distress was also found to be a risk factor that increases an individuals’ possibility of being involved in bullying by 115.0%. Moderate levels of psychological distress, on the other hand, put students at a 93.0% higher risk of being involved in bullying, followed by low levels of psychological distress which heightened the possibility by 39.0%.

Under the domain which addressed peer factors, students who identified as having a few or not many friends had a 31.0% and 8.0% lower chance of bullying involvement, respectively. Those who claimed to have no friends at all, however, had a 1.54 (95% CI = 0.56–0.86) times higher risk of being involved in bullying. When addressing quarrels and fights, it was found that those who quarrelled or fought with their friends were more prone to being involved in bullying. Those who always quarrelled increased their risk of being involved in bullying by 112.0%, followed by those who sometimes quarrelled and, lastly, by those who rarely quarrelled who increased their chances of bullying involvement by 85.0% and 51.0%, respectively.

From the data collected about school factors, it was discovered that individuals who acknowledged that they were mischievous or troublemakers in class had a 1.52 (95% CI = 1.06–2.06) times higher chance of being involved in bullying. Those who had no affection towards their teachers were also at a higher risk of being involved in bullying, which is 53.0% higher than those who do. Additionally, adolescents who rarely receive compliments from their teachers were included among those who had a higher risk of being involved in bullying, as they had a 1.49 (95% CI = 1.69–1.94) times higher chance than those who were always complimented by teachers. Students who had a majority of friends who never or rarely are helpful in the classroom had a 92.0% and 71.0% higher chance of bullying involvement, respectively. While those who sometimes received help from the majority of their friends within the classroom had a 21.0% chance of being involved in bullying. Students who skipped more than three classes in the past year had a 2.91 (95% CI = 2.90–1.72) times higher possibility of being involved in bullying when compared to those who never skipped classes.

## 4. Discussion

### 4.1. Prevalence of Bullying Involvement

The prevalence of bullying cases remains a significant problem globally. This study found that 79.1% of the respondents were involved in bullying as either bullies, victims, or bully–victims. This number is relatively high, given that only a small percentage of students were not involved in any way. When observed further, the percentage of students who were found to be victims was 16.3%, which is fairly close in range to the national average of 17.9%, as reported by the National Health and Morbidity Survey (Institute of Public Health, 2015), which was carried out in Malaysia. The numbers, however, differ from those obtained by other Southeast Asian countries, as well as globally [28].

Additionally, the current study found that the prevalence of bullies was at 14.4%. This finding differs from similar studies conducted in other countries, such as the study conducted in Lara, Venezuela, among 7th to 9th graders, a South African study conducted among 5074 8th to 11th graders from 72 schools in Cape Town and a Nigerian study conducted among 300 middle schoolers from Benin. The prevalence rates obtained by those studies ranged from 8.2% to 71.0% [29,30,31]. A past study in Malaysia, on the other hand, found a 49.2% prevalence rate of bullies [32]; therefore, the results obtained by the current study are considered relatively low. The prevalence of bully–victims was found to be the highest, with 48.4% of respondents identifying as having played both roles. Similar studies such as that among 2238 and 22,877 secondary school students from China, a South Korean study among 1756 secondary school students and a Turkish study among 372 teenagers produced prevalence rates between 2.6% to 15.0% [33,34,35,36].

In Malaysia, a past study among students from Kuala Lumpur shows that the prevalence of bullying victims was 17.6% [37], which was higher than that found in the current study. However, the important fact is that this group of students face the most problems and experience significant negative effects [38]. The variation in findings pertaining to the prevalence of bullying involvement can be explained by numerous factors. Among those is the fact that the questionnaires used were different in past studies and the characterization of what makes a bully, victim or bully victim differed from one another. Past findings largely relied on the structure of the questionnaires used, how each role was defined and measured, as well as the group of respondents studied. However, there is a significant difficulty in comparing and interpreting data pertaining to bullying, based on schools, community, juvenile or correctional institutions [39] and nations [40]. An influencing factor could be that students from different nations tend to have different perceptions regarding what can be categorized as bullying and how it varies from other forms of aggressive behaviour.

Secondly, students were likely to have had difficulties in reporting bullying involvement, resulting in errors in the findings. Additionally, there has yet to be information obtained from other relevant parties such as teacher or parents, which can be used as a reference as it may provide a clearer picture of bullying cases [41]. Lastly, another influencing factor could be the absence of students during data collection periods [42]. This would negatively influence the result obtained, given that data were collected during school hours.

### 4.2. Types of Bullying

Types of bullying can be categorized as verbal, relational, physical [43,44] and cyber-based bullying [45]. In this study, verbal bullying was found to be the most common (50.9%), followed by physical bullying (33.6%), relational bullying (10.6%) and cyberbullying (4.9%). The same pattern was observed among bullying victims, whereby the most frequent type of bullying they experienced as victims, was verbal bullying (38.8%), followed by relational bullying (30.6%), physical bullying (20.7%) and, lastly, cyberbullying (9.9%). The findings echo that of a study conducted among 2758 adolescents in Thailand [46] and another study conducted in 33 European countries, with respondents ages 11, 13 and 15 [44].

A study involving adolescents in Istanbul, Turkey, showed that 18.0% of the respondents were bullies and 27.0% were victims of cyberbullying [36]. It was a lot lower than the rates discovered by [47], which were 24.0% and 37.0% for bullies and victims, respectively. Additionally, when compared to 20 other countries in a study regarding the prevalence of cyberbullying cases, Malaysia is placed at the 17th spot [47]. According to the study, the awareness and knowledge regarding cyberbullying among Malaysians is relatively low in comparison to other countries, due to the lack of policies in schools that encourage the need to introduce programs related to cyberbullying prevention [47]. Furthermore, a practice of impoliteness communication strategy is considered common among youngsters in Malaysia as reported by Shaari and Kamaluddin [48].

Continuous involvement of students in bullying cases is closely linked to significant negative effects [49]. Bullying victims are generally associated with depression, anxiety, agoraphobia, panic disorders, low self-esteem [50,51,52], psychosomatic problems such as headaches, abdominal pains, sleep disturbances and low appetite [13]. The effects that it has on the bullies, on the other hand, include a 4 times higher chance of being convicted of crime in adulthood [53] and the chances have a possibility of increasing if the individual experiences severe psychiatric symptoms [54]. Other long-term consequences are related to anti-social behaviour and sexual predation [52,55]. Most bullies, on the other hand, hold more positive belief about the use of aggression and perceived bullying as a powerful survival tool [39].

In addition to bullies and victims, witnesses of school bullying are also at risk of developing severe mental health problems. The problems include depression, frequent worrying, drug abuse [56], persistent fear and feeling helplessness [57], as well as suicidal ideation [56]. Fear and the prospect of having to face the possible retaliation by the bully prevents witnesses from seeking help from others. This is evident given that bullying mostly occurs in the presence of observers and it was found that 80.0% of bullying cases were witnessed by others [1].

### 4.3. Factors Related to Bullying Involvement

When compared to 16-year-olds, adolescents aged 13 were 1.38 times more likely to be involved in bullying. This finding echoes the results obtained by the study conducted among adolescents in Thailand and Sri Lanka [46,58]. Younger age groups are generally smaller in terms of physical stature, less mature and have a lower stress tolerance level. Bullies tend to take advantage of this situation and target and victimize those younger than them as the victims are not capable of defending themselves. Therefore, it increases the chances of younger individuals’ bullying involvement.

In terms of gender, male students were found to have a 1.73 times higher risk of being involved in bullying situations. This supports the findings by a previous study involving Korean adolescent respondents, as well as a study with American respondents from Illinois [35,59]. Male adolescents’ involvement in bullying is significantly higher than that of female adolescents, especially as bullies and bully–victims. This could be because of the male adolescents’ physiological factors, such as hormonal influences which make them more prone to aggression than female adolescents.

From the ethnic perspective, Chinese adolescents were 55.0% less likely to be involved in bullying, followed by Malay adolescents who had a 44.0% less chance and Indian adolescents’ whose possibility of being involved in bullying is 34.0% lower than adolescents who belong to “Other” ethnic groups. These findings require further investigation as there are too few studies with which to make comparisons.

The differences in terms of culture relating to the perceptions of students, as well as their willingness to report the incidents, could have also affected the results obtained. However, it is important to acknowledge that the current findings do not provide any proof to suggest any specific bullying involvement based on ethnicities. However, it is possible that being of the majority or minority race might influence whether an individual plays the role of the bully, victim or both in bullying scenarios [37].

In addition to the factors discussed above, time spent on social media was also found to be significantly linked to students’ bullying involvement. Through this study, it was found that students who spent 1 to 4 h or more on a weekday and more than 4 h during the weekend were 1.43 and 1.28 times more likely to be involved in bullying, respectively. Despite being insignificant after other variables were taken into account, social media usage during weekdays should be considered as being important and a variable in future studies. It is possible that parents allow the children more social media access during the weekend to make use of the long hours available for well-needed rest at that point of the week.

This result mimics that of the study conducted by Pew and American Life Project consisting of 935 adolescent respondents, which found that the risk of being involved in cyberbullying was higher among individuals who had active profiles on social media platforms and spent more time on it [60]. Another case-control study was carried out among 13- to 17-year-old adolescents from Missouri, in the United States and the results indicated that students who were exposed to cyberbullying as either a cyberbully, a cyber-victim or both spent more time on social activities involving the computer [61].

Early data pertaining to the subject suggest that intervention strategies targeted at managing cyberbullying should focus on internet usage patterns, time spent on social media, activities an individual engages in on social media, as well as the characteristics of social media used by the adolescents. The exposure of students to risk factors that lead to involvement in cyberbullying needs to be further investigated.

The level of psychological distress is among the most important component that needs to be evaluated. It measures the mental health capacity of students. The current study found that experiencing any level of mental distress puts a student in a position where they are more prone to be involved in bullying in comparison to their peers who do not experience mental distress at all. This is consistent with the findings of other studies [62,63,64], which suggest that mental health distress heightens with increasing severity of bullying involvement.

The types of bullying involvement that have been highlighted within this study could lead to detrimental effects on adolescents’ mental health. As a result, adolescents may engage in anti-social behaviours such as smoking and consuming alcohol and have a possibility of participating in criminal behaviour in the future [65]. Moreover, bullying involvement has also been found to negatively impact student’s academic performance and excellence [46], in addition to their self-esteem [66].

Data obtained thus far show that adolescent bullying involvement is increasing globally [43]. Therefore, the failure to identify the distress level experienced by students could effectively lead to worrying outcomes. Such outcomes could range from suicidal ideation to other anti-social behaviour [32]. Therefore, mental health and emotional support need to be provided to adolescents who have been involved in bullying situations.

Additionally, it is important that future studies should investigate and take into account reports from other individuals within close proximity of adolescents, such as parents and peers to ease the process of identifying students who experience mental distress. Through this method, appropriate early intervention can be made possible and should be made an important endeavour by relevant authorities with the intention to produce adolescents who have strong mental capacities.

#### 4.3.1. Peer Factor

Behaviours that bring about risk to physical well-being, such as fights or other physically aggressive behaviour involving peers, are significantly linked to involvement in bullying cases. Studies have found that the higher the frequency of involvement in such behaviours, the higher the possibility for the involved student to also be involved in incidences of bullying, with chances that range from 51.0% to 112.0%. Other studies also echo the dose-response relationship between the number of bullying involvement incidences and adolescents’ aggressive behaviour.

A survey relating to peer bullying cases and problem behaviour among 921 Israeli students aged 13 to 15, revealed that peer relationship had a significant influence over those behaviours, whereby adolescents who have unhealthy and weak relationships with their peers are at increased risk of being involved in problems relating to aggressive behaviour in comparison to their other peers [67]. Other studies that set out to study the same relationship, among 2249 Venezuelan adolescents and 7338 Filipino adolescents, also produced results stating that there is a significant relationship between peer relationship and bullying involvements. Those who were involved in bullying were also reported to have been more prone to engaging in physical fights and vice versa, compared to their peers who were not involved in bullying [68,69]. Moreover, there also a significant relationship between motives of aggression (proactive and reactive) and bullying involvement. The relationship can be seen in a study involving 3359 students, which was conducted in Kota Palu, Indonesia [70]. In general, adolescents who are involved in bullying situations tend to lack the social skills required to build and maintain relationships with their peers [55]. Despite being powerful and highly capable, they lack strong internal characteristics [71].

Overall, the current study shows that adolescents’ bullying involvement is closely linked to peer aggression [38]. Therefore, to prevent other students from experiencing negative consequences and to preserve optimal adolescent health, it is of importance that more holistic approaches are taken to address bullying [2]. Further research should also address the development of psychometric scales to measure bullying behaviour. Additionally, it is of utmost importance that longitudinal studies be carried out, with the aim of studying bullying among students and related long-term protective factors.

#### 4.3.2. School Factor

Improving the relationship between teachers and students plays an important, positive and lasting influence in order to improve academic performance, as well students’ social behaviour [72]. In this study, teacher’s attitudes towards students act as a protective factor that could possibly reduce bullying involvement by 49.0% to 53.0%. Students who always build close and positive relationships and receive strong support and guidance, have a higher chance and tendency to improve their social skills, in comparison to students who experience conflicts and problems in the relationship they have with their teachers [73]. This suggests that a close and positive relationship with their teachers would indirectly and significantly reduce behavioural problems among adolescents, especially in terms of bullying involvement [74].

On the other hand, if the quality of a student-teacher relationship is compromised, it would lead to an increase in the rate of behavioural problems among students [75]. Students who have a negative relationship with their teacher cannot always control their emotions and tend to behave aggressively and impulsively. This opens them up to a heightened risk of being involved in bullying [73]. Additionally, these characteristics can become more severe when teachers always express anger towards these adolescents. In such a situation, bullying could become a norm in the classroom, in addition to negatively impacting the students’ academic performance and dampening their socio-emotional control [75]. The characteristics of the classroom also significantly influence the students’ involvement in bullying situations [76]. In a study in Finland, which involved almost 7000 students from 378 different classrooms, it was found that 87.0% of bullying involvement was predicted by factors relating to the environment within the classroom [77] and their behaviour within the classroom. In the same study, it was shown that students who were mischievous in the classroom had a 52.0% higher probability of being involved in bullying.

In addition, students who perceived that they had many friends within the classroom and that the friends were always helpful and supportive when needed had a 92.0% lower chance of being involved in bullying [76]. This shows that classroom factors can either promote or prevent bullying from taking place. The situation within the classroom can be described using the term “classroom norms”, which are closely linked to bullying [76]. In past social psychology literature, this norm was defined as being a rule, value or standard that is practised by a social group with/and behavioural characteristics that are perceived as relevant and appropriate within the group [78]. In this situation, the social group is students within a classroom. When observed closely, classroom norms can produce a descriptive index that describes the extent of student involvement in bullying within the classroom [79]. In reference to this fact, Ref. [80] showed that high rates of reported bullying within the classroom are closely linked to students’ tendency to enjoy bullying. Additionally, when bullying occurs continually, it influences observers to join in on bullying other students. As explained by [81], a norm in bullying involvement is that the bully is likely to be a popular student and the victim a less popular student who is frequently maltreated within the classroom. Regardless of the situation outside of the classroom, identifying students who are at risk of becoming bullies within the classroom and intensively educating them could reduce the student’s bullying involvement.

In identifying students’ sense of belonging in school, the frequency of a student’s intentional absence from educational periods was studied. It was found that the possibility of being involved in bullying rose with the frequency of truancy from school, making these students up to nearly 3 times more likely to become participants in bullying situations in comparison to others. The result echoes the findings put forth by a study in Beijing, China, which stated that bullying is linked to failure in school and high rates of truancy [82,83].

The degree of sense of belonging was also investigated and it was found that students who were involved in bullying tend to dislike their school and do not have an interest in joining activities relating to the school. This is supported by the result of studies conducted in Japan and Australia which found that a low sense of belonging in school is significantly correlated to students’ involvement in bullying [84,85]. Therefore, it is crucial to stress the need for the introduction of more activities that can foster students’ sense of belonging in school, as a method of intervention to manage cases of bullying. However, it is also important to identify the efficacy of the introduced programs to ensure that they can fulfil the set objective. It can be concluded that, in terms of the school environment, a lack of attention and awareness from relevant authorities, as well as a low level of involvement in school activities, can potentially contribute to a student’s involvement in bullying. This, in turn, creates a negative environment within the school and strains the relationship among students, especially when students perceive the school authorities as having failed in effectively managing bullying cases. Therefore, it is possible that interventions at the school level could prevent adolescents from being involved in bullying.

According to Ref. [72], the school’s intervention brings about the best outcomes, based on a few principles. Firstly, safety at school (including behaviour about cases of bullying and aggression) is a crucial dimension that requires attention in school settings [86]. Secondly, students need to understand that school have personnel whom always show support towards students who experience bullying [87]. A specific intervention by school social workers and counsellors is vital in addressing bullying among school children [88]. Acceptance towards the implementation of school social work intervention in addressing bullying issues has been found to be promising. School social worker involvements is essential in the education sector to reduce social problems, particularly bullying among school children. Lastly, the improvement of the social climate in schools have been proven to show a significant decrease in bullying cases in schools [53].

### 4.4. Other Relevant but Insignificant Factors

#### 4.4.1. Individuals

In a study within the population of adolescents in Chennai, India, it was found that nearly 78.0% of respondents owned smartphones or other electronic devices. This factor was considered to be significantly associated with bullying involvement, primarily cyberbullying [85]. However, while the current study uncovered a similar percentage of adolescents who own electronic devices, this variable was not found to be significantly related to the respondents’ involvement in bullying. Despite that, involvement in bullying, especially cyberbullying, has become a growing worry among adolescents across the globe [47]. Adolescents today have easier access to the internet via smartphones and other modern communication devices. This gives them the chance to directly harm other individuals without being easily discovered by the victim [89].

There is also proof of a negative gradient among adolescents who were involved in bullying, especially in the aspect of education [23,90]. In terms of academic functioning, students involved in bullying were found to have lower academic abilities and achievements [82]. However, in the current study, no significant relationship was found between academic performance and bullying involvement. Despite that, it is still necessary for further research to study the impact of academic performance, in addition to formulating better intervention strategies targeted at managing bullying behaviours.

#### 4.4.2. Family

Many past studies have identified a significant relationship between family and an adolescent’s bullying involvement. Those studies had found that adolescents from single-parent households and those with parents who had lower levels of education and lower job status were more prone to be involved in bullying [91,92,93]. The results cannot be compared or discussed regarding the current study as the study did not find a significant relationship between family factors and the respondents’ bullying involvement. Additionally, other studies also pointed out that a lower household income was significantly related to bullying involvement [94]. However, the current study also did not echo this finding.

Parents’ low education levels translate into low-status jobs that come with unstable or low incomes. It eventually translates into their intellectual abilities and specific knowledge which may not entirely adhere to norms and values of problem-solving [95]. Education levels can also be linked to parenting behaviours and, consequently, the child’s social skill development and strategies when faced with future challenges [96,97]. The relationship between single-parent homes and an adolescent’s risk of being involved in bullying can be explained through the lens of the lack of time the parent spends on interacting with their children, due to the fact that most of their time is spent on jobs and making ends meet. This can result in the parent having very little control over the adolescent and leave little room to discuss daily challenges or problems faced by the child. This could have a link to peer relationships. Alternatively, the influence of single parents can be explained by the stress that exists in a family which is neither stable nor complete. This is because stress experienced by parents and their lack of well-being is known to have a negative impact on the behaviour of adolescents [98,99].

## 5. Conclusions

Bullying among adolescents is a form of peer abuse and is defined as a set of negative behaviour and systematic abuse of power which involves repetition, harm and the presence of unequal power between the perpetrator and the victim. The findings of the study have identified important information. It shows that age, gender, ethnicity, time spent on social media, level of psychological distress, number of friends, frequency of domestic conflict with friends, presence of troublemaker student in classroom, student affection towards their teachers, frequency of compliments from teachers, attitude of students in helping their friends and practice of intentionally skipping classes are the most influential factors associated to students’ involvement in bullying.

The use of a survey in the study allowed students the opportunity to increase their awareness of bullying cases in school, the types of bullying and the impacts of it on weaker students. Without that kind of awareness among students and teachers within a school, a holistic approach to address bullying cannot be fulfilled in Malaysian educational institutions. The message regarding the experience and feelings of adolescents when involved in bullying is crucial in the first steps of creating awareness in society. Additionally, the findings will allow relevant authorities to tackle the problem via more specific and strategic methods, enabling the effective management and prevention of bullying within the nation.

The study also provides the chance for us to observe the responses of students regarding anti-bullying programs that are already in place in the long term. However, it can only be done if there is an appropriate research method and structure in place, in addition to the use of valid statistical analyses. Having considered this, the researchers carefully selected the choice of method used to ensure that it is suitable and could measure what they had aimed to. Moreover, the findings can also provide the school authorities to build a clearer understanding of the behaviours and reactions of students concerning bullying involvement.

The study shows that adolescent involvement in bullying is a phenomenon that is quite widespread in Malaysian secondary schools, with factors in domains related to the individual and school being significantly related to an adolescent’s bullying involvement. Peer factors, on the other hand, were found to have a minimal level of significance in their relationship with bullying involvement, despite being significant influences still. The findings from the current study suggest that there is a need for drastic bullying intervention strategies to be employed in schools, with a focus on students with a higher risk of being involved in bullying and continuous, multilevel assessments that determine and ensure the effectiveness of the programs employed. The information obtained from the students has substantially helped the researchers understand the nature and prevalence of bullying in school, as well as the ways through which these students were affected. Furthermore, the findings provide important preliminary information that helps improve understanding about the issues pertaining to bullying cases within schools and can act as a reference when evaluating anti-bullying policies and the actions that can be taken by school authorities to curb this phenomenon. In addition, the information obtained can be used by relevant authorities in the Ministry of education, Ministry of Health and Royal Police of Malaysia as a guide to determine necessary courses of actions.

## Figures and Tables

**Table 1:** Distribution of individual factors (*n* = 4469).

Factor	Characteristics	*n* (%)
Age		
	13	1374 (30.7)
	14	1652 (37.0)
	16	1443 (32.3)
Gender		
	Male	2135 (47.8)
	Female	2334 (52.2)
Ethnicity		
	Malay	2895 (64.8)
	Chinese	835 (18.7)
	Indian	392 (8.8)
	Other	347 (7.7)
Academic Performance		
	Excellent	833 (18.6)
	Good	2136 (47.8)
	Satisfactory	1126 (25.2)
	Not Satisfactory	374 (8.4)
Time spent on social media per day on a weekday (hours)		
	Never	857 (19.2)
	<1	1517 (33.9)
	1–4	1482 (33.2)
	>4	613 (13.7)
Time spent on social media during the weekend (hours)		
	Never	527 (11.8)
	<1	1130 (25.3)
	1–4	1591 (35.6)
	>4	1221 (27.3)
Own a personal handphone		
	Yes	3584 (80.2)
	No	885 (19.8)
Own an iPad, iPod, tablet, personal computer		
	Yes	2974 (66.5)
	No	1495 (33.5)
Frequency of overall internet usage		
	Never	214 (4.8)
	<once/month	301 (6.7)
	1–2/month	450 (10.1)
	1–2/week	984 (22.0)
	Almost Everyday	2520 (56.4)
Time spent on the internet on a weekday (hours)		
	Never	597 (13.4)
	<1	1566 (35.0)
	1–4	1442 (32.3)
	>5	864 (19.3)
Time spent on the internet during the weekend (hours)		
	Never	327 (7.3)
	<1	1064 (23.8)
	1–4	1499 (33.5)
	>5	1581 (33.4)
Level of psychological distress (mental disturbance)		
	None	1087 (24.3)
	Slightly	1393 (31.2)
	Moderate	1141 (25.5)
	Bad	848 (19.0)

**Table 2:** Distribution of peer factors (*n* = 4469).

Factor	Characteristics	*n* (%)
Number of friends		
	Many	3474 (77.7)
	A few	691 (15.5)
	Not Many	263 (5.9)
	None	41 (0.9)
Close friends		
	Yes	4109 (91.9)
	No	359 (8.1)
Frequency of quarrels/fights with friends		
	Always	220 (4.9)
	Sometimes	1392 (31.1)
	Sometimes	2220 (49.7)
	Other	637 (14.3)
Frequency of time spent with friends outside of schooling hours		
	Always	797 (17.8)
	Sometimes	1849 (41.4)
	Sometimes	1376 (30.8)
	Others	447 (10.0)

**Table 3:** Distribution of Family Factors (*n* = 4469).

Factor	Characteristics	*n* (%)
Father’s education level		
	Primary	330 (7.4)
	Secondary	2693 (60.2)
	Tertiary	1084 (24.3)
	Other	362 (8.1)
Mother’s education level		
	Primary	260 (5.8)
	Secondary	2834 (63.4)
	Tertiary	1069 (23.9)
	Other	306 (6.8)
Father’s career level		
	Professional	1654 (37.0)
	Non-professional	2257 (50.5)
	Retiree	2834 (8.2)
	Other	191 (4.3)
Mother’s career level		
	Professional	1214 (27.2)
	Non-professional	1277 (28.6)
	Retiree	126 (2.8)
	Other	1852 (41.4)
Household income		
	<RM 1000	587 (13.1)
	RM 1000–RM 5000	2539 (56.8)
	RM 5001–RM 10,000	927 (20.7)
	>RM 10,000	416 (9.4)
Parents’ marital status		
	Married	4064 (90.9)
	Divorced	276 (6.2)
	Other	129 (2.9)
Time spent with father in a day (hours)		
	1–6 h(s)	2271 (50.8)
	7–10 h	875 (19.6)
	>10 h	746 (16.7)
	Other	577 (12.9)
Time spent with mother in a day (hours)		
	1–6 h(s)	1537 (34.4)
	7–10 h	1017 (22.8)
	>10 h	1665 (37.3)
	Other	250 (5.5)
Parental help with child’s homework		
	Always	772 (17.3)
	Sometimes	2061 (46.1)
	Sometimes	1113 (24.9)
	Other	523 (11.7)
Parents’ awareness about their child’s whereabouts		
	Always	3147 (70.4)
	Sometimes	957 (21.4)
	Sometimes	265 (5.9)
	Never	100 (2.3)
Fierce arguments between parents		
	Always	243 (5.5)
	Sometimes	1217 (27.2)
	Sometimes	2075 (46.4)
	Never	934 (20.9)
Frequency of sharing their feelings with their father		
	Always	562 (12.6)
	Sometimes	1302 (29.1)
	Sometimes	1202 (26.9)
	Other	1403 (31.4)
Frequency of sharing their feelings with their mother		
	Always	1500 (33.6)
	Sometimes	1546 (34.6)
	Sometimes	829 (18.6)
	Other	594 (13.2)
Arguments between student and their parents		
	Always	149 (3.3)
	Sometimes	665 (14.9)
	Sometimes	1135 (25.4)
	Never	2519 (56.4)
Overprotective parents		
	Always	1482 (33.2)
	Sometimes	1711 (38.3)
	Sometimes	951 (21.3)
	Never	325 (7.3)
Immature parenting by parents		
	Always	1262 (28.2)
	Sometimes	1545 (34.6)
	Sometimes	1160 (26.0)
	Never	502 (11.2)
Presence of siblings		
	Yes	4169 (93.3)
	No	300 (6.7)
Number of older siblings		
	1–4	2894 (64.8)
	>4	366 (8.2)
	None	1209 (27.0)
Number of younger siblings		
	1–4	2895 (64.8)
	>4	175 (3.9)
	None	1399 (31.3)
Number of household members		
	<2	144 (3.2)
	2–4	1643 (36.8)
	>4	2682 (60.0)

**Table 4:** Distribution of school factors (*n* = 4496).

Factor	Characteristics	*n* (%)
Interest in attending school		
	Yes	3930 (87.9)
	No	539 (12.1)
Love towards school		
	Yes	3833 (85.8)
	No	636 (14.2)
Presence of at least one person for the students to share their feelings with, in school		
	Yes	3196 (71.5)
	No	1273 (28.5)
Respect and affection towards their teacher		
	Yes	4063 (90.9)
	No	406 (9.1)
Frequency of appraisal by teacher		
	Always	1497 (33.5)
	Sometimes	2013 (45.0)
	Sometimes	703 (15.7)
	Never	256 (5.7)
Mischievous/troublemaker in classroom		
	Yes	507 (11.4)
	No	3962 (88.6)
Majority of friends are helpful in the classroom		
	Always	1596 (35.7)
	Sometimes	2025 (45.3)
	Sometimes	673 (15.1)
	Never	175 (3.9)
Sense of belonging in school		
	Always	1742 (39.0)
	Sometimes	1431 (32.0)
	Sometimes	652 (14.6)
	Never	644 (14.4)
Frequency of intentionally skipping class		
	Once	628 (14.1)
	2–3	589 (13.3)
	>3	286 (6.4)
	Never	2960 (66.2)
Frequency of absence from school		
	1–15	3066 (68.6)
	15–25	235 (5.3)
	>25	164 (3.6)
	Never	1004 (22.5)

**Table 5:** Individual factors related to bullying involvement.

Factors		Raw OR	95% CI	ᵡ^2^ stat.(df) ^a^	*p* Value
Ethnicity					
	Malay	0.74	0.55; 1.01	3.70 (1)	0.049
	Chinese	0.59	0.43; 0.83	9.57 (1)	0.019
	Indian	0.49	0.34; 0.70	15.12 (1)	<0.001
	Other	1.00			
Gender					
	Male	1.85	1.51; 2.03	54.69 (1)	<0.001
	Female	1.00			
Academic Performance					
	Excellent	1.00			
	Good	0.81	0.66; 0.90	4.43 (1)	0.038
	Satisfactory	0.97	0.77; 1.22	0.07 (1)	0.792
	Unsatisfactory	1.67	1.18; 2.36	8.25 (1)	0.039
Time spent on social media on a weekday (hours)					
	<1	0.87	0.71; 1.06	1.96 (1)	0.162
	1–4	1.28	1.05; 1.58	5.67 (1)	0.017
	>4	2.10	1.57; 2.80	25.29 (1)	<0.001
	Never	1.00			
Time spent on social media on a weekend (hours)					
	<1	1.03	0.82; 1.31	0.81 (1)	0.776
	1–4	1.34	1.07; 1.68	6.48 (1)	0.011
	>4	2.56	1.98; 3.30	51.57 (1)	<0.001
	Never	1.00			
Frequency of overall internet usage					
	Never	1.00			
	<once/month	1.00	0.67; 1.50	0.02 (1)	0.999
	1–2/month	1.25	0.85; 1.82	1.26 (1)	0.262
	1–2/week	1.01	0.72; 1.42	0.01 (1)	0.943
	Almost everyday	1.50	1.08; 2.07	5.90 (1)	0.015
Time spent on the internet on a weekday (hours)					
	<1	0.90	0.67; 1.06	2.19 (1)	0.139
	1–4	1.20	0.95; 1.51	2.34 (1)	0.126
	>4	2.10	1.53; 2.67	24.87 (1)	<0.001
	Never	1.00			
Time spent on the internet on a weekend (hours)					
	<1	0.97	0.73; 1.28	0.06 (1)	0.811
	1–4	1.19	0.90; 1.56	1.47 (1)	0.225
	>4	2.25	1.69; 2.90	31.17	<0.001
	Never	1.00			
Level of psychological distress (mental disturbances)					
	Good	1.00			
	A little	1.47	1.23; 1.76	17.67 (1)	<0.001
	Moderate	2.25	1.84; 2.76	61.52 (1)	<0.001
	Bad	3.54	2.76; 4.55	98.19 (1)	<0.001

Raw OR = Raw Odd Ratio; CI = Confidence Interval; df = degree of freedom. ^a^ Likelihood-Ratio Test (LR).

**Table 6:** Characteristics of peer factors related to bullying involvement.

Factors		Raw OR	95% CI	ᵡ^2^ Stat.(df) ^a^	*p* Value ^a^
Number of friends					
	Many	0.44	0.16; 1.23	2.48 (1)	0.115
	Few	0.30	0.11; 0.85	5.10 (1)	0.024
	Not Many	0.38	0.13; 1.11	3.10 (1)	0.079
	None	1.00			
Have close friends					
	Yes	0.72	0.54; 0.96	4.87 (1)	0.027
	No	1.00			
Frequency of quarrels/fights with friends					
	Always	3.86	2.47; 6.05	34.98 (1)	<0.001
	Sometimes	2.60	2.10; 3.23	73.26 (1)	<0.001
	Sometimes	1.81	1.49; 2.20	36.10 (1)	<0.001
	Other	1.00			
Frequency of time spent with friends after school hours					
	Always	2.10	1.53; 2.69	24.52 (1)	<0.001
	Sometimes	1.58	1.25; 2.00	14.55 (1)	<0.001
	Sometimes	1.29	1.01; 1.64	4.17 (1)	<0.001
	Other	1.00			

Raw OR = Raw Odd Ratio; CI = Confidence Interval; df = degree of freedom. ^a^ Likelihood-Ratio Test (LR).

**Table 7:** Characteristic of family factors associated with bullying involvement.

Factors		Raw OR	95% CI	ᵡ^2^ Stat.(df) ^a^	*p* Value ^a^
Father’s education level					
	Primary	0.86	0.59; 1.25	0.67 (1)	0.425
	Secondary	0.80	0.61; 1.10	2.37 (1)	0.124
	Tertiary	1.05	0.72; 0.85	8.67 (1)	0.039
	Other	1.00			
Father’s level of occupation					
	Professional	1.38	0.97; 1.97	3.13 (1)	0.077
	Not professional	1.04	0.74; 1.48	0.06 (1)	0.815
	Retiree	1.22	0.80; 1.86	0.86 (1)	0.353
	Other	1.00			
Household income					
	< RM 1000	1.23	1.04; 1.60	5.57 (1)	0.018
	RM 1000–RM 5000	1.17	0.92; 1.50	1.70 (1)	0.198
	RM 5001–RM 10,000	1.73	1.30; 2.38	11.04 (1)	0.001
	>RM 10,000	1.00			
Parental help with homework					
	Always	1.00			
	Sometimes	1.12	0.92; 1.37	1.36 (1)	0.244
	Rarely	1.46	1.16; 1.82	10.57 (1)	0.001
	Other	1.23	0.94; 1.61	2.28 (1)	0.131
Parents’ knowledge about respondent’s whereabouts					
	Always	1.00			
	Sometimes	1.40	1.16; 1.69	12.58 (1)	<0.001
	Rarely	1.36	0.97; 1.88	3.36 (1)	0.067
	Never	2.14	1.17; 3.94	6.03 (1)	0.014
Frequency of intense arguments between parents					
	Always	1.83	1.24; 2.70	9.306 (1)	0.002
	Sometimes	1.45	1.18; 1.79	11.97 (1)	0.001
	Rarely	1.06	0.88; 1.27	0.37 (1)	0.546
	Never	1.00			
Frequency of intense arguments between respondent and parents					
	Always	2.04	1.26; 3.30	8.45 (1)	0.004
	Sometimes	1.89	1.49; 2.38	27.55 (1)	<0.001
	Rarely	1.30	1.09; 1.56	8.97 (1)	0.003
	Never	1.00			
Overprotective parents					
	Always	1.40	1.06; 1.86	5.45 (1)	0.021
	Sometimes	1.35	1.03; 1.79	4.55 (1)	0.033
	Rarely	1.02	0.76; 1.36	0.01 (1)	0.909
	Never	1.00			

^a^ Likelihood-Ratio Test (LR).

**Table 8:** Characteristics of school factors related to bullying involvement.

Variable		Raw OR	95% CI	ᵡ^2^ Stat.(df) ^a^	*p* Value ^a^
Student’s interest in attending school					
	Yes	1.00			
	No	3.52	1.53; 2.61	25.94 (1)	<0.001
Student’s love towards the school					
	Yes	1.00			
	No	2.21	1.79; 2.84	37.17 (1)	<0.001
Presence of at least 1 person the students can share their feelings within the school					
	Yes	1.00			
	No	1.22	1.03; 1.43	5.42 (1)	0.022
Affection towards their teacher					
	Yes	1.00			
	No	2.49	1.79; 3.47	29.17	<0.001
Frequency of appraisal from teacher					
	Always	1.00			
	Sometimes	1.23	1.05; 1.44	6.37 (1)	0.012
	Rarely	1.65	1.31; 2.10	17.95 (1)	<0.001
	Never	1.59	1.12; 2.25	6.73 (1)	0.009
Mischievous/Troublemaker in classroom					
	Yes	2.76	2.03; 3.75	42.20 (1)	<0.001
	No	1.00			
Majority of peers are helpful in the classroom					
	Always	1.00			
	Sometimes	1.40	1.19; 1.64	17.91 (1)	<0.001
	Rarely	1.98	1.56; 2.52	31.46 (1)	<0.001
	Never	3.48	2.06; 5.89	21.60 (1)	<0.001
Sense of belonging in school					
	Always	1.00			
	Sometimes	1.10	0.93; 1.30	1.30 (1)	0.254
	Rarely	1.69	1.33; 2.15	18.27 (1)	<0.001
	Never	1.52	1.20; 1.92	12.34 (1)	<0.001
Frequency of skipping class					
	Once	2.04	1.61; 2.59	34.44 (1)	<0.001
	2–3	2.33	1.81; 3.01	42.42 (1)	<0.001
	>3	5.40	3.28; 8.88	44.20 (1)	<0.001
	Never	1.00			
Frequency of truancy from school (days)					
	1–15	1.24	1.05; 1.46	6.24 (1)	0.013
	15–25	2.90	1.86; 4.53	21.90 (1)	<0.001
	>25	2.68	1.61; 4.46	14.29 (1)	<0.001
	Never	1.00			

Raw OR = Raw Odd Ratio; CI = Confidence Interval; df = degree of freedom, ^a^ Likelihood-Ratio Test (LR).

**Table 9:** Collinearity diagnosis via variance inflation factor.

Factor	Standardized Coefficient No		*p* Value	95% CI	Collinearity Statistics	
	B	Error Std.			Tolerance	VIF
Constants	0.997	0.081	<0.001	0.84; 1.16		
Age	−0.008	0.005	0.097	−0.02; 0.01	0.978	1.023
Gender	−0.093	0.012	<0.001	−0.12; −0.07	0.953	1.050
Ethnicity	0.002	0.006	0.713	−0.01; 0.02	0.973	1.028
Time spent on the internet during weekends	0.019	0.006	0.02	0.01; 0.03	0.989	1.012
Level of psychological distress	0.060	0.006	<0.001	0.05; 0.07	0.933	1.071
No. of friends	−0.029	0.010	0.005	−0.05; −0.01	0.956	1.046
Frequency of fights with friends	−0.051	0.008	<0.001	−0.07; −0.04	0.941	1.062
Household incomes	0.017	0.007	0.019	0.03; 0.05	0.989	1.011
Mischievous/Troublemaker in class	0.061	0.019	0.002	0.02; 0.09	0.919	1.089
Affection towards their teacher	−0.062	0.021	0.004	−0.11; −0.02	0.913	1.096
Frequency of appraisal by teacher	0.024	0.007	0.001	0.01; 0.04	0.933	1.072

Error Std = Error Standard; CI = Confidence Interval; VIF = Variance Inflation Factor.

**Table 10:** Hosmer dan Lemeshow test.

Chi-Square	df	*p* Value
6.069	8	0.640

df = degree of freedom.

**Table 11:** Classification Table.

Observed		Predicted		
		Dichotomous Overall Outcome Logic		Actual Percentage
Overall dichotomous outcome		Not Involved	Involved	
	Not Involved	156	779	16.7
	Involved	112	3415	96.8
Total percentage				80.0

**Table 12:** Determinant factors of bullying involvement.

Variable		Adj. OR	95% CI	ᵡ^2^ Stat. (df) ^a^	*p* Value ^a^
INDIVIDUAL					
Age					
	13	1.38	1.12; 1.70	9.31 (1)	0.002
	14	1.13	0.93; 1.37	1.53 (1)	0.217
	16	1			
Gender					
	Male	1.73	1.47; 2.04	42.17 (1)	<0.001
	Female	1.00			
Ethnicity					
	Malay	0.66	0.47; 0.91	6.49 (1)	0.011
	Chinese	0.45	0.31; 0.65	18.18 (1)	<0.001
	Indian	0.56	0.38; 0.84	7.91 (1)	0.005
	Others	1.00			
Time spent on social media on weekends (hours)					
	<1	1.09	0.84; 1.42	0.40 (1)	0.528
	1–4	1.43	1.09; 1.87	6.87 (1)	0.009
	>4	1.28	1.69; 3.10	28.53 (1)	<0.001
	Never	1.00			
Level of psychological distress					
	Good	1.00			
	Mild	1.39	1.14; 1.69	11.03 (1)	0.001
	Moderate	1.93	1.55; 2.40	34.51 (1)	<0.001
	Severe	2.55	1.94; 3.34	45.66 (1)	<0.001
PEER					
No. of friends					
	Many	1.00			
	A Few	0.69	0.56; 0.86	11.21 (1)	0.001
	Not Many	0.92	0.65; 1.30	0.24 (1)	0.625
	None	1.54	0.50; 4.58	0.54 (1)	0.463
Frequency of quarrels/fights with friends					
	Always	2.12	1.24; 3.26	8.08 (1)	0.004
	Sometimes	1.85	1.45; 2.36	24.54 (1)	<0.001
	Rarely	1.51	1.21; 1.86	13.66 (1)	<0.001
	Other	1.00			
SCHOOLS					
Mischievous/Troublemaker in class					
	Yes	1.52	1.06; 2.06	5.35 (1)	0.021
	No	1.00			
Affection towards their teacher					
	Yes	1.00			
	No	1.53	1.06; 2.20	5.14 (1)	0.023
Frequency of compliments from teachers					
	Always	1.00			
	Sometimes	1.16	0.97; 1.38	2.66 (1)	0.103
	Rarely	1.49	1.16; 1.94	9.34 (1)	0.002
	Never	1.27	0.86; 1.89	1.45 (1)	0.228
Majority of friends are very helpful in class					
	Always	1.00			
	Sometimes	1.21	1.02; 1.44	4.66 (1)	0.031
	Rarely	1.71	1.24; 2.09	12.45 (1)	<0.001
	Never	1.92	1.09; 3.38	5.13 (1)	0.024
Intentionally skipping class (frequency)					
	Once	1.67	1.30; 2.15	16.04 (1)	<0.001
	2–3	1.63	1.24; 2.14	12.27 (1)	<0.001
	>3	2.91	2.90; 1.72	16.04 (1)	<0.001
	Never	1.00			

Raw OR = Raw Odd Ratio; CI = Confidence Interval; df = degree of freedom ^a^ Likelihood-Ratio Test (LR).

## Data Availability

The data presented in this study are available on request from the corresponding author. The data are not publicly available due to data confidentiality imposed by Malaysian Education Ministry.
